# Selective colony area method for heterogeneous patient-derived tumor cell lines in anti-cancer drug screening system

**DOI:** 10.1371/journal.pone.0215080

**Published:** 2019-04-17

**Authors:** Jang Ho Cho, Ju-Sun Kim, Seung Tae Kim, Jung Yong Hong, Joon Oh Park, Young Suk Park, Do-Hyun Nam, Dong Woo Lee, Jeeyun Lee

**Affiliations:** 1 Division of Hematology-Oncology, Department of Medicine, Samsung Medical Center, Sungkyunkwan University School of Medicine, Seoul, Korea; 2 Department of Neurosurgery, Samsung Medical Center, Sungkyunkwan University School of Medicine, Seoul, Korea; 3 Department of Biomedical Engineering, Konyang University, Daejeon, Korea; University of South Alabama Mitchell Cancer Institute, UNITED STATES

## Abstract

We aimed to establish a fluorescence intensity-based colony area sweeping method by selecting the area of highest viability among patient-derived cancer cells (PDC) which has high tumor heterogeneity. Five gastric cancer cell lines and PDCs were screened with 24 small molecule compounds using a 3D micropillar/microwell chip. 100 tumor cells per well were immobilized in alginate, treated with the compounds, and then stained and scanned for viable cells. Dose response curves and IC_50_ values were obtained based on total or selected area intensity based on fluorescence. Unlike homogeneous cell lines, PDC comprised of debris and low-viability cells, which resulted in an inaccurate estimation of cell viability using total fluorescence intensity as determined by high IC_50_ values. However, the IC_50_ of these cells was lower and accurate when calculated based on the selected-colony-area method that eliminated the intensity offset associated with the heterogeneous nature of PDC. The selected-colony-area method was optimized to accurately predict drug response in micropillar environment using heterogeneous nature of PDCs.

## Introduction

Despite advances in targeted therapy and immunotherapy for solid cancer, one of the most challenging problems in oncology is that development of active drugs is still a slow multi-layered, complicated process. Considering the time consumption, high cost, and low success rate of pre-clinical and clinical development of oncology drugs, more efficient and accurate platforms for oncology drug screening are urgently needed.

The activity of oncology drugs has been studied in two-dimensionally (2D) cultured cancer cell lines. However, it has been long challenged that these preclinical model systems minimally reflect the *in vivo* microenvironment [1–6] and low probability for translating into clinical benefit in cancer patients [7, 8]. In order to better recapitulate actual patient’s tumor, three-dimensional (3D) cell culture systems had been suggested in the past decade as an alternative preclinical tumor models. Studies on integrating 3D experimental environment with high-throughput screening methods are ongoing with some success, including our previously described system [9–19].

Patient-derived tumor cells are attractive as effective tools for preclinical evaluation of personalized medicine strategies [20–24], even though these models are limited due to their cost, and tumor heterogeneity [20, 25, 26]. Unlike established, immortalized cell lines that are a homogeneous population clearly distinguishable from dead cells or colonies with low viability, the patient-derived cancer cells are usually heterogeneous comprising dead cells or cells with low viability and robust tumor cells. When assessed using a 3D cell-based compound screening system, debris or patient-derived cancer cells with low viability exhibited low fluorescence intensity; however, the high number of these low intensity dots had a cumulative effect on the total intensity within alginate spots, resulting in an intensity offset. In the present study, we sought to address this issue by setting a florescence intensity threshold on the same field of an alginate spot and calculating the differences in intensity. As proof of concept, five gastric cancer cell lines and patient-derived cancer cells were screened with 24 compounds (S1 Table) based on this selected-colony-area method in micropillar high throughput system.

## Materials and methods

### Cell lines and culture conditions

Human gastric cancer cells (MKN-28, MKN-45, MKN-74, SNU-216, SNU-484, SNU-601, SNU-638, SNU-668, SNU-719, and AGS) were purchased from the Korean Cell Line Bank (Seoul, South Korea). All cell lines were cultured in RPMI 1640 medium (Gibco) supplemented with 10% foetal bovine serum. Cell lines were maintained at 37°C in a 5% CO_2_-humidifed atmosphere and passaged every four days.

### Patient-derived tumor cell culture

Table 1 summarizes the baseline characteristics of the patients. Patient-derived cancer cells were collected most commonly from ascites. Malignant ascites were collected from patients as previously described with informed consent [9–11, 18]. The collected effusions (1–5 L) were divided into 50 mL tubes, centrifuged at 1500 rpm for 10 min, and washed twice with phosphate-buffered saline (PBS). Cell pellets were suspended in culture medium and plated onto 75 cm^2^ culture flasks. Cell lines and PDCs were grown to 80–90% confluency and passaged using TrypLE Express (Gibco BRL) and seeded with 3D culture medium consisting of DMEM F/12 supplemented with 10 mM HEPES, 1% antibiotic-antimycotic solution, 2% 50× B27, 1% 20× N2, 1% 100× Glutamax (Gibco BRL), 10 mM human gastrin I, 1 mM N-acetyl-L-cysteine (Sigma Aldrich), 10 μg/mL insulin, 20 ng/mL basic fibroblast growth factor (bFGF), and 50 ng/mL EGF (PeproTech).

**Table 1:** Baseline clinical features of patient-derived cancer cells.

No.	Cancer types	Date of collection	Age year	Sex	Source of PDCs	ECOG	Pathology	Stage
PDC#1	Pancreatic Cancer	2014-11-19	65	M	Ascites	1	Ductal adenocarcinoma	IV
PDC#2	Pancreatic Cancer	2014-08-13	48	F	Ascites	1	Adenocarcinoma, moderately differentiated	IV
PDC#3	Pancreatic Cancer	2014-10-14	39	F	Ascites	1	Ductal adenocarcinoma	IV
PDC#4	Gastric cancer	2016-10-11	58	M	Stomach	1	Tubular adenocarcinoma, poorly differentiated	IV
PDC#5	Gastric cancer	2014-11-11	70	F	Ascites	1	Tubular adenocarcinoma, poorly differentiated	IV

### Chip layout and experimental procedure

**Fig 1:**
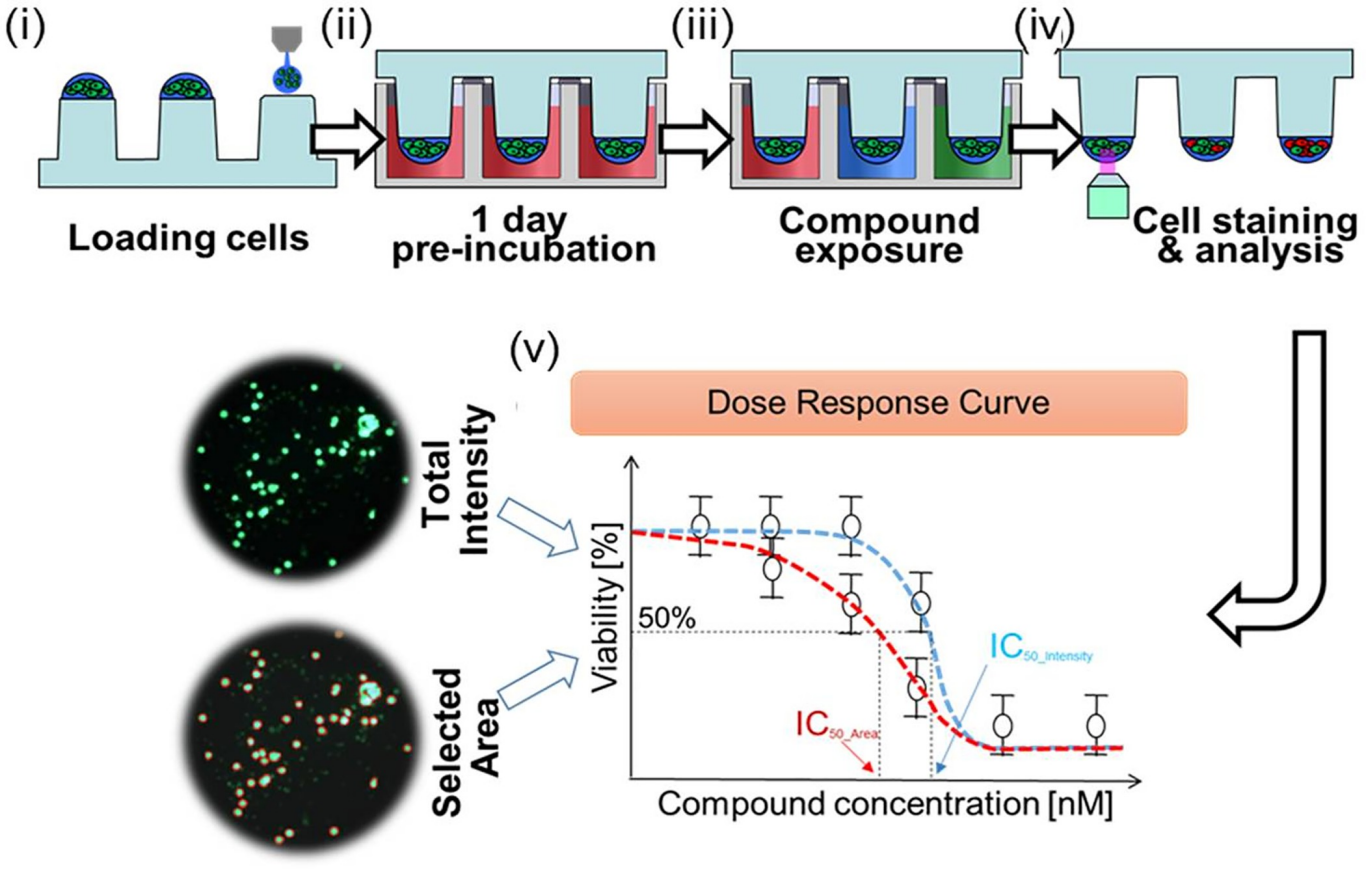
Schematic view of 3D cell based micropillar/microwell chip platform for high-throughput screening and experimental procedure. (i) Cells are dispensed and immobilized in alginate onto the top of the micropillars and (ii) dipped in the microwells containing growth media for 1-day culture by sandwiching the micropillar and microwell chips. (iii) Compounds are dispensed into the microwells and cells are exposed to the compounds by moving the micropillar chip to a new microwell chip. (iv) 3D-cultured cells are stained with Calcein AM, and the dried alginate spot on the micropillar chip is scanned for data analysis. (v) Dose response curve and IC_50_ calculation based on total intensity and selected area.

The basic layout of the micropillar/microwell chip for a 12-compound screening was shown in our previous study [12]. The microwell chip was divided into 12 regions with each region further divided into a 6 × 6 microwell array. A single dose-response curve for each compound was obtained per region. Each region tested 6 compound concentrations that included one control well and five different dosages. For compound analyses, as shown as **Fig 1-i**, approximately 100 cells in 50 nL with a 0.5% alginate concentration by volume (0.5w/w) were automatically dispensed onto a micropillar chip by using ASFASpotter ST (Medical & Bio Device, South Korea). The ASFA Spotter ST uses a solenoid valve (The Lee Company, USA) for dispensing the 50 nL droplets of the cell–alginate mixture and 950 nL of media or compound. After dispensing the cells, as shown as **Fig 1-ii**, the micropillar chip containing cell lines and PDCs in alginate was sandwiched (or “stamped”) with the microwell chip for 3D cell culture and compound efficacy tests. After 1 day of incubation at 37°C to stabilize the cells, as shown in **Fig 1-iii**, the micropillar chip containing the cells was moved to a new microwell chip filled with various test compounds. A single chip can screen 12 compounds for 6 replicates. Next, the combined chips were incubated for 3 days (cell lines) or 5 days (PDCs). After 3 or 5 days, cell viability against the compounds was measured with Calcein AM live cell staining dye (4 mM stock from Invitrogen), which stains viable cells with green fluorescence. The staining dye solution was prepared by adding 1.0 μL of Calcein AM (4 mM stock from Invitrogen) in 8 mL staining buffer (MBD-STA50, Medical & Bio Device, South Korea). For staining, as shown **Fig 1-iv**, micropillar chips were moved into a staining buffer in Calcein AM for 45 minutes. The stained chip was washed for 30 minutes in the staining buffer solution and then dried completely in a dark environment. To measure cell viability quantitatively after staining the alginate spots, cells on the micropillar chip were scanned using optical scanner (ASFAScanner ST, Medical & Bio Device, South Korea) shown in **Fig 1-iv**. Scanned images were evaluated using an image analysis software (ASFA Ez SW, Medical & Bio Device, South Korea) shown in **Fig 1-v**. **Fig 1** shows a schematic representation of the experimental procedure described above.

### IC_50_ calculation

Cell viability values were normalized to their corresponding control wells (no drug treatment), because not all control conditions exhibited 100% cell viability. The sigmoidal dose-response curves (variable slope) and IC_50_ values (i.e., concentration of the compound resulting in 50% inhibition of cell growth) were obtained with the following equation:

$$Y=Bottom+\left[\frac{Top-Bottom}{1+10^{(\log\;IC50-X)\times n_{H}}}\right]$$ (1)

where IC_50_ is the midpoint of the curve; *n*_*H*_ is the hill slope; *X* is the logarithm of the compound concentration, and *Y* is the response (cell viability). The ASFAScanner ST (Medical & Bio Device, South Korea) software sets the *Bottom* as zero and the *Top* as 100% when the data are fit to a curve.

### Ethical statement

This investigation was conducted in accordance with the ethical standards of the Declaration of Helsinki and national and international guidelines and was approved by the Institutional Review Board at Samsung Medical Center in Seoul, Korea (IRB No. 2015-10-062 & 2011-07-089).

## Results

### Colony area sweeping

To validate fluorescence intensity-based colony area sweeping method, five patient-derived cancer cells (Table 1) were screened with 24 compounds. Fig 1 shows a representative alginate spot containing multiple colonies that were imaged using both the total and threshold-based fluorescence intensity methods. Dose response curves of the gastric cancer cell lines and patient-derived cancer cells were generated for each of the 24 compounds screened to compare the two methods of determining cell viability.

**Fig 2:**
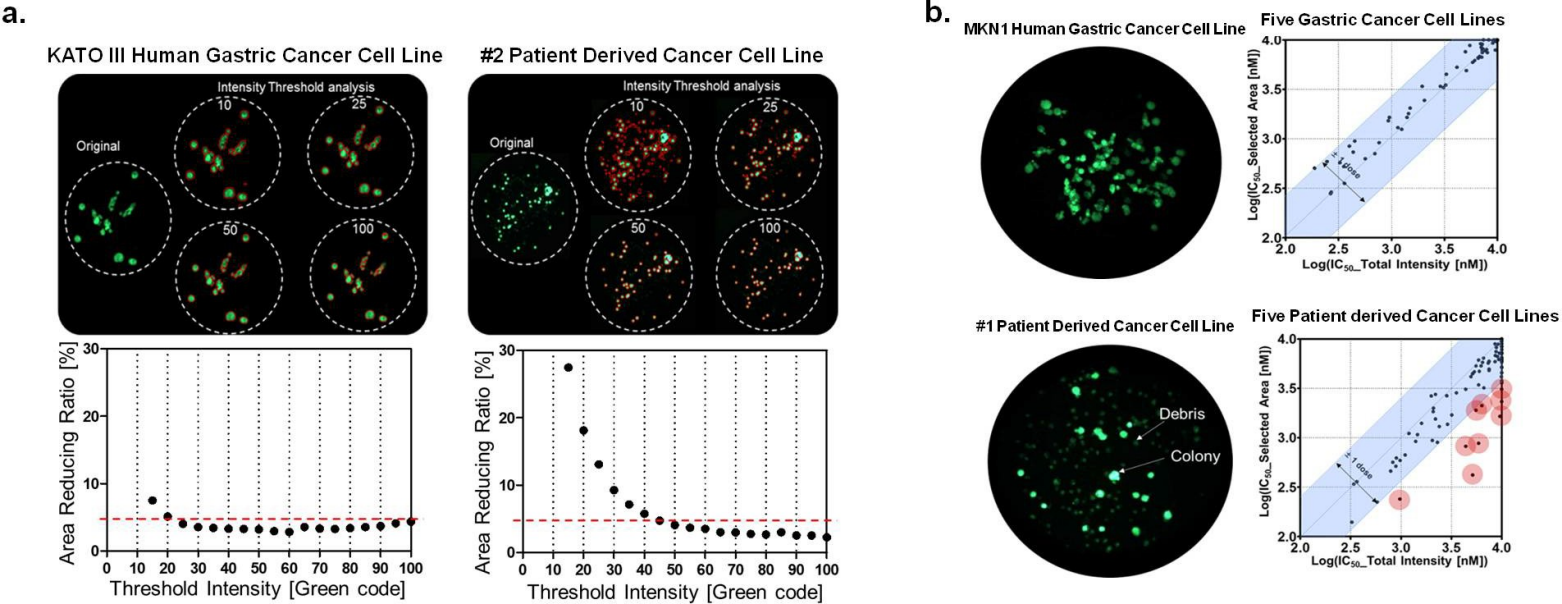
**(a) Cell images and area reducing ratio of KATO III human gastric cancer cell line and #2 patient-derived cancer cell line (PDC) according to intensity thresholds.** Red mark in the images are the selected colonies according to intensity thresholds. **(b) Colony images and IC**_**50**_ **values comparison between MKN1 human gastric cancer cell line and #1 PDC.** Contrary to MKN1 gastric cancer cell line image, colony formation was seen only with some of the cells from the #1 PDC. In the five PDCs, IC_50_ values calculated by the selected area were generally lower than those calculated using total intensity. Ten outliers (red circles) who IC_50_ difference between total intensity and selected area analysis is more than 1 dose (3 times) were observed.

Fig 2A shows cell images of KATO III human gastric cancer cells and patient-derived cancer cell sample #2. The majority of KATO III human gastric cancer cells formed colonies, but the patient-derived cancer cell sample #2 formed only few colonies. In the KATO III human gastric cancer cell line, the reducing ratios were very small in low intensity thresholds (10~30), because most colonies had high cell viability and there were no debris and cells with low viability. The other four gastric cancer cell lines showed similar results. However, patient-derived cancer cell sample #2 had high amount of debris and cells with low viability, therefore its reducing ratio was high (greater than 5%) in low intensity thresholds (10~30). The same sample displayed low reducing ratios (less than 5%) when intensity thresholds were greater than 45. Images of the patient-derived cancer cell sample #2, as shown in Fig 2A, displayed multiple faint green dots that indicated cell debris and low cell viability. By sweeping the colony area using an intensity threshold, the optimum total colony area was determined when the reducing ratio was less than 5%. Using this method with threshold intensities set to 50 and 100, images of patient-derived cancer cell sample #2 successfully selected only highly viable colonies, while eliminating the debris and cells with low viability. The other four patient-derived cancer cells showed similar results.

### Comparison of IC_50_ in the gastric cancer cell lines and PDCs

Fig 2B shows representative images of the gastric cancer cell line, MKN1, and patient-derived cancer cells and compares the IC_50_ values calculated using the total intensity with the selected colony area method. Colony formation was seen with most MKN1 cells, but only with some of the cells from the patient-derived cancer cell line #1 (Fig 2B). In the five patient derived cancer cell lines, all 120 IC_50_ values (5 cell lines and 24 drugs), calculated using both methods, were similar between the cancer cell lines (Table 2). The difference in IC_50_ values between the methods was within 1 dose (3 times), suggesting no significant differences between the two methods. However, for the five patient-derived cancer cell samples, IC_50_ values calculated by the selected colony area method were generally lower than those calculated using total fluorescence intensity. In IC_50_ calibration by total intensity, the intensity offset increased cell viability in a high dosage of the drug. Specifically, we observed 10 outliers with large differences of more than 1 dose (3 times) (Fig 2B).

**Table 2:** IC_50_ of Pancreatic and Gastric cancer patient derived cancer cells (PDCs).

Drug	IC_50_ of 5 pancreatic and gastric cancer PDCs (uM)									
	PDC #1		PDC #2		PDC #3		PDC #4		PDC #5	
	Total intensity	Selected area	Total intensity	Selected area	Total intensity	Selected area	Total intensity	Selected area	Total intensity	Selected area
1_Olaparib	10	10	10	6.3	2.4	1.3	9.2	8.9	10	10
2_AZD4547	10	7.8	0.9	0.5	4.4	0.8	5.2	4.5	10	3.8
3_AZD5363	10	6	7.5	5	1	0.6	1.6	1.4	7.5	4.8
4_Volitinib	10	10	10	10	10	10	10	10	10	6.5
5_Selumetinib	10	7.3	0.8	0.6	2.3	0.9	8.6	8.9	9.1	6.7
6_AZD 1775	10	10	6.3	5.3	1.1	0.7	2.2	2.8	5.6	5.3
7_Everolimus	10	8.5	10	10	10	10	2	2.7	10	10
8_Crizotinib	10	8.4	5.9	6.1	8.6	5.9	5.4	4.7	10	4.6
9_Dasatinib	10	7.1	6.7	3.2	1.4	0.9	7.4	5.7	10	6.4
10_Regorafenib	10	10	9.5	7.2	10	10	10	9.5	10	5.9
11_LJM716	10	10	10	10	10	10	10	10	10	10
12_Vemurafenib	10	6.2	5.9	3.4	2.1	2	6	4.7	6.5	5.8
13_Cetuximab	10	7.2	10	2.3	3.2	1.7	10	10	10	6.2
14_GDC0449	10	10	10	10	10	10	10	9	10	10
15_Blind drug A	10	10	10	10	10	10	8.9	8.2	10	10
16_Dacomitinib	6.4	2.1	0.6	0.2	0.8	0.5	4.4	3.1	5.9	0.9
17_Lapatinib	10	6.1	5.6	1.9	2.2	1.6	10	9.6	10	8.4
18_BEZ235	8.8	7.5	9.6	1.7	6.6	6.4	1	0.2	10	3.1
19_AZD2014	10	10	10	10	10	10	1.2	1.1	10	10
20_LEE011	10	10	10	10	10	10	10	10	10	10
21_Staurosporin	7.5	6	0.3	0.3	0.4	0.4	0.3	0.1	5.2	0.4
22_Neratinib	10	10	8.7	6.8	2.1	0.9	0.9	0.6	10	5.2
23_BGJ398	10	3.6	2.9	1.4	2.9	2.7	5	4.2	10	4.2
24_Blind drug B	9.4	6.2	2.1	1.8	1.5	1.1	3.6	2.9	10	5.2

**Fig 3:**
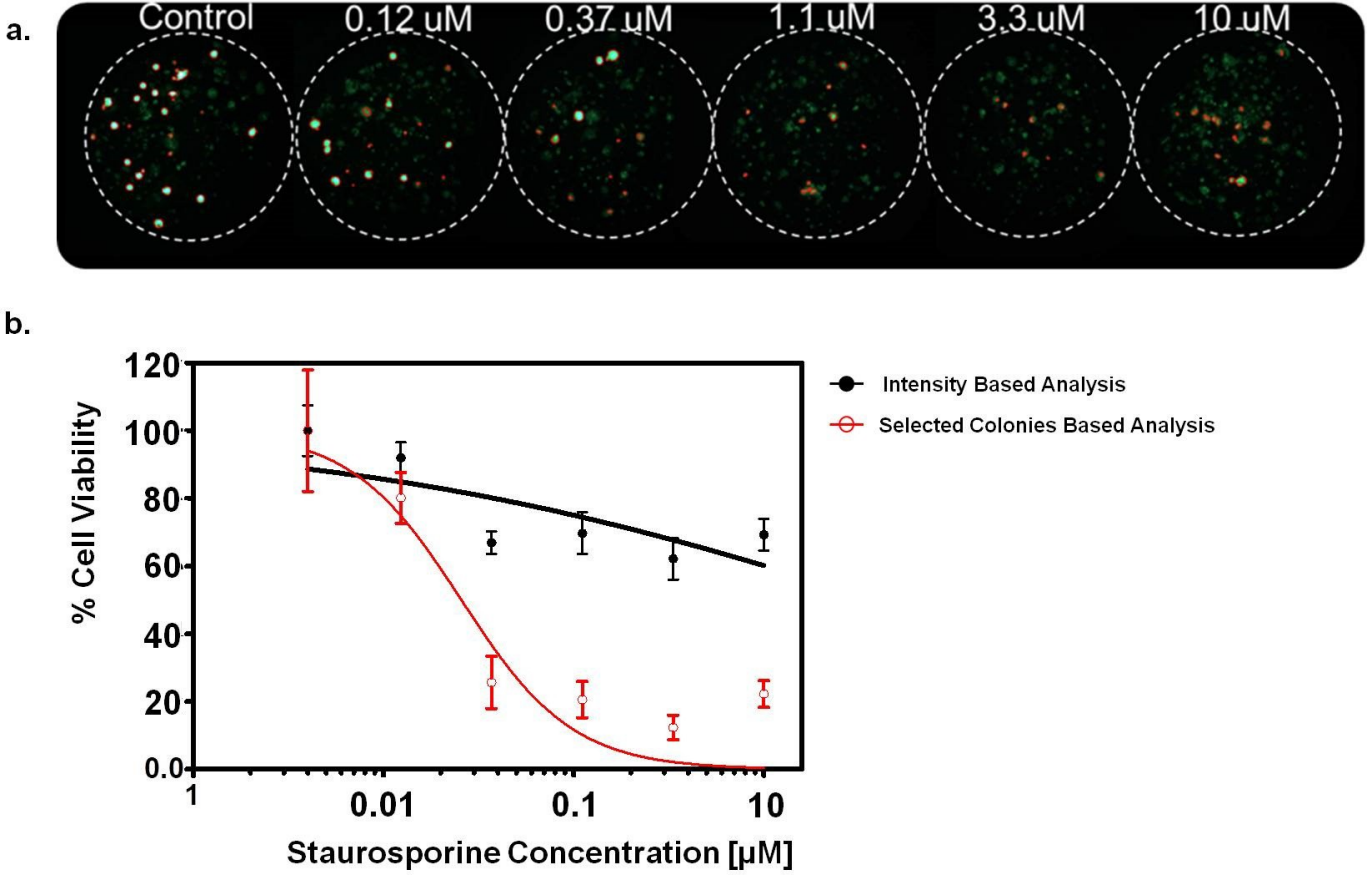
**Alginate spot images (a) and dose response curve (b) of #5 patient-derived cancer cell line with staurosporine.** (a) Red marks in alginate spot images are the selected colonies by the optimal intensity threshold. The image of an alginate spot in 10 uM staurosporine showed 8% of viable cells, while the control with established cell line has 100% cell viability. (b) Even though 10 uM staurosporine affected cell viability of #5 PDC, high amount of debris and cells with low viability resulted in a large intensity offset and increased cell viability in the dose response curve. But, in the selected colony area method, the cell viability was reduced to 30% upon 10uM staurosporine treatment compared to the control.

Fig 3 shows the alginate spot images and dose response curve of one example among the 10 outliers. As shown in Fig 3A, the image of an alginate spot harbouring patient-derived cancer cell line #5 in 10 μM staurosporine showed faint dots and 8% of viable cells, while the control alginate spot with established cell line has bright colonies and 100% cell viability. The fluorescence intensity of the test condition was 80% of the intensity of the control condition, leading to an IC_50_ value that was very high even though staurosporine exerted considerable inhibitory effect on patient-derived cancer cell line #5. To solve this problem, we removed the intensity offset by selecting a colony area of high viability using an intensity threshold. The black and red lines represent the dose response curves with cell viability calculated using total fluorescence intensity and the selected colony area, respectively. Even though cell viability of patient-derived cancer cell line #5 was affected by 10 μM staurosporine, the debris and cells with low viability produced a large intensity offset and artificially increased the cell viability calculation in the dose response curve (Fig 3B). The viability using only highly viable colonies was reduced to 30% upon 10 μM staurosporine treatment which is comparable to the control. Therefore, for patient-derived cancer cell line with heterogeneous cell population and varying cell viability within colonies, the selected-colony-area method in micropillar high throughput system reflected better for drug screening.

## Discussion

Patient-derived cancer cells are more representative of the *in vivo* tumor microenvironment. In the present study, we examined the potential of using a fluorescence intensity-based colony area sweeping method for high throughput quantitative analysis of 3D-cultured patient-derived cancer cells as a novel approach in predicting drug responses in these cells. By selecting a high viability colony area using an intensity threshold, we could successfully eliminate the intensity offset and solve the problem associated with the heterogeneous nature of patient-derived cancer cells. To the best of our knowledge, this is the first study to address this tumor heterogeneity issue.

Gastric cancer cell lines, which are a homogeneous population of cells, formed colonies in alginate spots without debris or cells of low viability. However, we found that 3D-cultured patient-derived cancer cells growing in alginate spots are a heterogeneous population with much debris or low-viability cells. To quantify cell viability, total intensity in an alginate spot was used in our previous work [12]. In case of patient-derived cancer cells, debris and low-viability cells produced an intensity offset, increasing the calculated IC_50_ values. As mentioned above, this intensity offset could be avoided by sweeping colony areas using a fluorescence intensity threshold and selecting for highly viable colonies. The optimal intensity threshold and the selected colony area were determined by calculating the reducing ratio of the selected colony area according to the fluorescence intensity thresholds ranging from 10 to 100 (Fig 2A**)**. These reducing ratios were reduced when the intensity threshold increased. When the total colony area was measured using a low intensity threshold, such as 10 or 25, the visible faint green dots artificially increase the selected colony area and increased the cell viability value. This increase accounted for the correspondingly high IC_50_ values in drug screening experiments.

The IC_50_ values determined by the selected colony area method were compared to those calculated using total intensity fluorescence. The 5 gastric cancer cell lines exhibited very similar IC_50_ values for both analyses, while the patient-derived cancer cell group exhibited 10 outliers (Table 2). These 10 outliers had a high intensity offset due to presence of dead and low-viability cells. The new method described here allowed us to correct the intensity offset and successfully measure the IC_50_ values in heterogeneous patient-derived cancer cell samples.

Ideal preclinical models should closely resemble real patient conditions regarding molecular profiles and clinical features. Moreover, well-established patient-derived cancer cells are useful to screen for and demonstrate the sensitivity of novel targeted agents [27]. However, unlike homogeneous cancer cell lines, patient-derived cancer cells consist of heterogeneous cell populations, even when they are from the same cancer from a single biopsy. Some populations upon culturing display a diverse distribution of colony size. Therefore, area-based analysis of the colonies is not enough to confirm total cell viability [16]. The method that we describe in our current study can successfully measure IC_50_ values in heterogeneous patient-derived cancer cells.

## Supporting information

S1 Table Twenty-four drugs screened for five gastric cancer cell lines and patient-derived cancer cell lines and the target genes of each drugs. (PDF)
