# Supplementary material for: Selective colony area method for heterogeneous patient-derived tumor cell lines in anti-cancer drug screening system
Source: PLoS One. 2019 Apr 17;14(4):e0215080. doi: 10.1371/journal.pone.0215080 (PMC6469764; doi:10.1371/journal.pone.0215080)
Supplement: S1 Table — (PDF) [file pone.0215080.s001.pdf]

S1 Table. Twenty-four drugs screened for five gastric cancer cell lines and patient-derived cancer cell lines and the target genes of each drugs.

Drugs	Target
1_Olaparib	PARP1/2
2_AZD4547	FGFR1/2/3
3_AZD5363	AKT1/2/3
4_Volitinib	MET
5_Selumetinib	MEK
6_AZD 1775	WEE-1
7_Everolimus	mTOR
8_Crizotinib	MET and ALK
9_Dasatinib	Abl, Src and c-Kit
10_Regorafenib	VEGFR, PDGFRβ, KIT, RET and RAF-1
11_LJM716	HER3 monoclonal antibody
12_Vemurafenib	B-Raf *v600E*
13_Cetuximab	EGFR monoclonal antibody
14_GDC0449	Hedgehog
15_Blind drug A	c-Met
16_Dacomitinib	EGFR
17_Lapatinib	EGFR and HER2
18_BEZ235	PI3K and mTOR
19_AZD2014	mTOR
20_LEE011	CDK4/6
21_Staurosporin	PKC (as used positive control)
22_Neratinib	HER2 and EGFR
23_BGJ398	FGFR1/2/3
24_Blind drug B	TrkA/B/C, ROS1 and ALK
